# *Staphylococcus aureus* on Sinus Culture Is Associated With Recurrence of Chronic Rhinosinusitis After Endoscopic Sinus Surgery

**DOI:** 10.3389/fcimb.2018.00150

**Published:** 2018-05-15

**Authors:** Anastasios Maniakas, Marc-Henri Asmar, Axel E. Renteria Flores, Smriti Nayan, Saud Alromaih, Leandra Mfuna Endam, Martin Y. Desrosiers

**Affiliations:** ^1^Centre de Recherche du Centre Hospitalier de l'Université de Montréal, Montreal, QC, Canada; ^2^Division of Otolaryngology-Head and Neck Surgery, Centre Hospitalier de l'Université de Montréal, Montreal, QC, Canada; ^3^Division of Otolaryngology-Head and Neck Surgery, McMaster University, Hamilton, ON, Canada; ^4^Department of Surgery, Cambridge Memorial Hospital, Cambridge, ON, Canada; ^5^Faculty of Medicine, King Saud University, Riyadh, Saudi Arabia

**Keywords:** chronic rhinosinusitis, *Staphylococcus aureus*, revision surgery, endoscopic sinus surgery, budesonide nasal irrigation

## Abstract

**Objectives:** Identify whether identification of *S. aureus* on conventional culture is a predictor of success or failure after ESS followed by budesonide nasal irrigations (BUD) in chronic rhinosinusitis (CRS) patients at high risk of recurrence.

**Methodology:** Prospective clinical trial including 116 patients from a tertiary care center at high-risk of disease recurrence following ESS+BUD. Blood samples, microbial swabs, and SNSS/SNOT-22 were taken on the day of surgery (Visit-1) and 4 months postoperatively (Visit-2). Outcomes were evaluated using symptoms and mucosal status as assessed by the Lund-Kennedy endoscopic score.

**Results:** Seventy-five patients (69.4%) attained SNOT-22 MCID or higher. (Mean = 33.4, range 9–75). Objective documentation of recurrence of disease, as defined by combined endoscopic/symptomatic criteria, was noted in 58/116 patients (50%). Revision surgery was associated with a significantly higher rate of disease recurrence (60.0 vs. 28.0%; *p* < 0.001). Culture for *Staphylococcus aureus* was associated with disease recurrence, preoperatively and at 4 months post-surgery (*p* = 0.020; *p* < 0.001). This was restricted to post-operative cultures in the revision group (10.0 vs. 48.8%; *p* < 0.001). Other factors associated with poor outcome included intolerance to non-steroidal anti-inflammatory drugs (NSAID) (*p* = 0.036). Significantly higher Lund-Kennedy scores in the recurrence groups despite similar symptom intensity, emphasizing the importance of considering objective outcome in addition to patient-reported ones.

**Conclusion:** Patients undergoing revision ESS are at high risk of disease recurrence, even when budesonide irrigations are used post operatively. Presence of *S. aureus* on culture pre-operatively or at 4 months post-ESS is associated with a negative outcome. This suggests that *S. aureus* negatively influences outcome, possibly via a number of mechanisms, including interactions with the (i) immune system, (ii) regeneration and repair of the sinus epithelium, or (iii) via interference with the sinus microbiome. This suggests that *S. aureus* may be a simple and inexpensive biomarker for disease severity and indicates a clear need to better appreciate *S. aureus* on how it contributes mechanistically to disease development and persistence in order to develop targeted therapeutic strategies.

## Introduction

Chronic rhinosinusitis (CRS) is characterized as a chronic inflammation of the paranasal sinuses and is considered to be one of the most frequent chronic inflammatory diseases with approximately 10% of the population currently affected (Chen et al., 2003; Hastan et al., 2011). Surgical therapy is common with more than 400,000 sinus surgeries performed per year in the United States alone (Anand, 2004). Unfortunately, up to one in three (DeConde and Soler, 2016) patients with CRS will fail endoscopic sinus surgery (ESS) and post-operative medical therapy, rendering them refractory to maximal medico-surgical treatment. Patients with refractory disease present with reduced quality of life, poor sleep, fatigue, and acute exacerbations of their CRS, resulting in significant increases in the economic burden of the disease (Rudmik et al., 2014), presently estimated at $10,000 annually per refractory patient, or 12.8 billion overall in the United-States alone.

Unfortunately, despite the high costs of CRS, its pathogenesis remains elusive, which stalls the development of novel therapeutic strategies. Authors have reported various factors associated with ESS failure, whether it be anatomical anomalies or associated medical comorbidities, amongst several other reported etiologies (Mendelsohn et al., 2011). Furthermore, our group and others have described disease parameters which may render a patient at a higher risk of revision ESS with maximal medical therapy failure, such as having had prior ESS for CRS, elevated serum IgE (≥150 kIU/L) and/or eosinophil levels (≥500 cells/mm), a young age (≤38) (Nader et al., 2010).

One of the principal issues is that little is known about the early events leading to the development of CRS. Monitoring patients prospectively following ESS may represent a means of improving our understanding. While in other respiratory diseases such as asthma and allergic disorders, birth cohorts which track a population from an early age have helped identify factors influencing development of disease (Jackson et al., 2017) we unfortunately do not yet have “birth cohorts” for CRS. However, monitoring sinus cavities after ESS may represent an opportunity. CRS is different from asthma and other atopic disorders as surgical therapy is available which can clear disease. Despite the fact that this post-ESS control may only be temporary, monitoring patients after ESS can almost be considered as a birth cohort since following surgery, we can profile changes in molecular mechanisms and microbiome as the healing cavity regenerates epithelium and repopulates its microbiome. These early post-ESS changes appear important to disease recurrence, as outcome appears relatively stable following the 6 month post-ESS time point (Rudmik et al., 2014). This setting may thus offer a privileged environment to identify factors associated with disease remission or recurrence.

We hypothesized that patients with prior sinus interventions and highly virulent pathogens such as Gram negative bacteria and *Staphylococcus aureus* would be associated with a poorer outcome post-ESS. In order to prospectively evaluate this hypothesis and further identify factors influencing disease development, we performed a clinical trial evaluating the post-ESS disease evolution of a group of CRS patients at high risk of recurrence following ESS, assessing epidemiological, clinical, biochemical and microbial outcomes following ESS for CRS.

## Materials and methods

### Study design and patient population

A prospective study was undertaken between November 2014 and December 2016 in our tertiary medical center to monitor post-ESS evolution in a cohort of CRS (with or without nasal polyposis) patients older than 18 years of age at a high-risk of disease recurrence. All surgeries were performed by the senior author (M.D.), who performed all of the post-operative management and assessments. This study was approved by the Ethics Review Board of the University of Montreal Health Center (Centre Hospitalier de l'Université de Montréal) CHUM-14.140.

All patients were recruited and informed written consent was obtained prior to the surgery. Recruitment was performed by a member of the research group not involved in the patient's care on the day of surgery on consecutive patients between the study dates who met inclusion criteria. Patients were included if they had at least one of the criteria used to qualify a patient at a “high-risk” of recurrence previously listed. Exclusion criteria included patients who had received topical or systemic antibiotic up to 4 weeks prior to the surgical intervention. In addition, patients with cystic fibrosis, inverted papilloma, osteoma, cystic masses, mucoceles, skull base lesions, or any other sinonasal tumors were excluded from the study.

All patients were managed with mucosal- and middle-turbinate sparing ESS as required for disease clearance, which usually involved complete sphenoethmoidectomy and frontal sinusotomy. Septoplasty was performed as required for access, usually using a targeted endoscopic technique. All patients received broad-spectrum antibiotics and an oral prednisone taper for 14 days following surgery. Patients were seen for cavity cleaning at 14 days (± 3 days), at which point post-operative once-daily nasal irrigations with 1.0 mg budesonide ampules (BUD) (Pulmicort Respules, AstraZeneca, Mississauga, ON, Canada) was initiated and continued throughout the rest of the 4-month observation period.

Assessments were performed on the day of surgery (Visit 1) and repeated at 4 months postoperatively (Visit 2).

### Patient evaluation

All patients had the following laboratory tests at recruitement and at 4 month follow-up: total IgE, High Specificity C-Reactive Protein (hsCRP) and complete blood count (CBC). Serum was also retrieved and preserved for future analyses at every visit. Patient-centered outcomes were assessed using the Sino-Nasal Symptom Score (SNSS) as well as the Sino-Nasal Outcome Test (SNOT-22) (Piccirillo et al., 2002) questionnaires. Endoscopic evaluation of the sinonasal cavities was performed at Visit 2 and scored using the modified Lund-Kennedy scoring system (Psaltis et al., 2014).

### Definition of disease recurrence vs. remission

In order to minimize variability of assessment due to varied patient perceptions of disease, we used a rigorous definition using endoscopic assessment of mucosal disease. We based this concept on the criteria used for diagnosis of CRS, where symptoms alone cannot be relied upon and objective evidence of disease must be present. We thus defined “disease remission” as absence of mucosal disease on endoscopy. Absence of disease was defined endoscopically as oedema ≤1 on the modified Lund-Kennedy mucosal grading scale. Any evidence of recurrence of polyposis was deemed to have recurrent disease.

### Bacteriology

Swab culture sampling was performed at the beginning of every ESS and at Visit 2 at the level of the ethmoid bulla using a thin aluminum wire swab with a mini-tip swab (BBL Cultureswab PLUS—BD Diagnostics Inc., Franklin Lakes, NJ) under direct rigid endoscopy. Care was taken to avoid contaminating the swab by touching the nasal vestibule or cavity wall. Samples were processed by the hospital laboratory where they were plated and streaked for aerobic bacteria isolation on a mannitol salt agar, a chocolate Haemophilus agar, and a MacConkey agar (Oxoid Inc, Nepean, ON). Anaerobic bacteria isolation was performed on a Brucella Agar with 5% Sheep blood and in a Schaedler Anaerobe Broth (Oxoid Inc, Nepean, ON).

### Statistical analyses

All data were tabulated using Microsoft Excel and all statistical analyses were performed using STATA 13.1 (STATACorp LP, College Station, TX). Quantitative variables are presented to describe patient medical and surgical history as well as associated pathologies. A two-tailed Pearson Chi-square or Fisher's exact tests were used to analyze the prevalence and proportion of demographic variables and specific bacterial strains between patients. SNSS and SNOT-22 scores as well as laboratory values amongst patients before and after ESS were evaluated using a two-sample Student *T*-test with unequal variances. A Pearson correlation coefficient was used to evaluate any clinically significant correlation between continuous variables. The differential relative abundance of any bacterial species between culture types was measured using a Wilcoxon signed-rank test. For all statistical analyses, a *p* < 0.05 was considered statistically significant.

## Results

Between November 2014 and December 2016, a total of 116 high-risk patients meeting our inclusion criteria, interested in participating and available for follow-up underwent ESS for CRSwNP or CRSsNP. Demographics are found in Table 1. Overall, patients responded well to ESS with a clinically and statistically significant mean decrease in twelve-point SNSS and SNOT-22 scores of 3.4 and 21.3, respectively. SNSS and SNOT-22 questionnaires demonstrated a strong correlation between each other, both at Visit 1 (*r* = 0.762; *p* < 0.001) and Visit 2 (*r* = 0.761; *p* < 0.001). Using the minimal clinically important difference (MCID) for the SNOT-22 of 9.5 (Browne et al., 2007), 75 patients (69.4%) attained SNOT-22 MCID or higher at 4 months (Mean = 33.4, range 9–75). Preoperative SNSS and SNOT-22 scores demonstrated a moderate correlation with postoperative Visit 2 SNSS and SNOT-22 scores (*r* = 0.385; *p* < 0.001 and *r* = 0.395; *p* < 0.001, respectively). Visit 2 SNOT-22 had a small correlation with Lund-Kennedy total scores (*r* = 0.236; *p* = 0.014), while Visit 1 SNSS and SNOT-22, and Visit 2 SNSS scores had no correlation with Lund-Kennedy Visit 2 scores.

**Table 1:** Patient demographics.

**Number of patients recruited**	**116**
Age (range)	49 (20–78)
Sex (Male:Female)	57 M:59 F
Asthma	75 (64.7%)
**Tobacco Use**	
Never	52 (44.8%)
Active	19 (16.4%)
Former	45 (38.8%)
Polyposis	108 (93%)
**Race**	
Caucasian	104 (89.7%)
Hispanic	6 (5.2%)
Asian	2 (1.7%)
Arabic	4 (3.4%)
Previous ESS	80 (69.0%)
Number of previous ESS (if applicable)	1.8 (0–5)
Allergies (all types)	90 (77.6%)
Seasonal	44 (37.9%)
NSAID	23 (19.8%)
Total IgE kIU/L	239.6 (0–1600)
Eosinophils cells/mm	431.6 (0–1390)

### Disease remission vs. recurrence

Using our strict criteria for disease, recurrence post ESS+BUD was significantly associated with patients undergoing revision ESS (*p* = 0.001), or with an allergy/intolerance to non-steroidal anti-inflammatory drugs (NSAID) (*p* = 0.036) (Table 2). While there was a trend toward higher failure rates in patients with asthma (*p* = 0.080) or CRS without polyposis (*p* = 0.061), this did not attain significance. No laboratory values were significantly different between patients having disease remission or recurrence post-ESS. Furthermore, there was no statistically significant difference between the “remission” and “recurrence” groups with regard to the SNSS and SNOT-22 scores at Visit 2 (*p* = 0.306 and *p* = 0.098, respectively).

**Table 2:** Disease remission vs. recurrence in all patients.

**Total: 116 patients**	**Remission**	**Recurrence**	***p*-value**
Patients	58	58	
Age	49.7	48.0	*p* = 0.490
Male	27 (46.6%)	30 (51.7%)	*p* = 0.577
Female	31 (53.4%)	28 (48.3%)	
Asthma	33 (56.9%)	42 (72.4%)	*p = 0.080*
Previous ESS	32 (55.2%)	48 (82.8%)	*p = 0.001*
Smoking			
Never	24 (41.4%)	28 (48.3%)	*p* = 0.591
Active	11 (19.0%)	8 (13.8%)	
Former	23 (39.6%)	22 (37.9%)	
Polyposis	57 (98.3%)	51 (87.9%)	*p = 0.061*
Race			
Caucasian	52 (89.7%)	52 (89.7%)	*p* = 1.000
Hispanic	3 (5.2%)	3 (5.2%)	
Asian	1 (1.7%)	1 (1.7%)	
Arabic	2 (3.4%)	2 (3.4%)	
Allergies (all types)	48 (82.8%)	42 (72.4%)	*p* = 0.182
Seasonal	27 (46.6%)	17 (29.3%)	*p = 0.056*
NSAID	7 (12.1%)	16 (27.6%)	*p = 0.036*
*S. aureus* (pre-ESS)	4 (7.3%)	14 (24%)	*p = 0.020*
*S. aureus* (post-ESS)	7 (13.0%)	24 (47.1%)	*p < 0.001*
*P. aeruginosa* (pre-ESS)	1 (1.8%)	5 (8.6%)	*p* = 0.207
*P. aeruginosa* (post-ESS)	1 (1.9%)	6 (11.8%)	*p = 0.056*
Gram negative (pre-ESS)	13 (23.6%)	12 (20.7%)	*p* = 0.706
Gram negative (post-ESS)	6 (11.1%)	13 (25.5%)	*p = 0.056*
SNSS (post-ESS)	4.4	5.2	*p* = 0.306
SNOT22 (post-ESS)	25.5	32.6	*p* = 0.098
Lund-Kennedy (post-ESS)	0.98	6.3	*p < 0.001*

Bacterial swabs demonstrated a statistically significant association between the presence of *S. aureus* preoperatively and disease recurrence following ESS+BUD at 4 months postoperatively. The presence of *S. aureus* at Visit 2 was also significantly associated with disease recurrence. In the group with disease recurrence, 14 patients had *S. aureus* preoperatively, of which 3 (21%) had a *S. aureus*-free microbial swab postoperatively, while there were 9 (64%) who were not able to clear it and an additional 15 neo-colonizations.

### Revision ESS patients: disease remission vs. recurrence

A subgroup analysis was performed looking at only patients undergoing revision ESS for CRS (*n* = 80) (Table 3). Of these, 35 (43.8%) had had more than one ESS for CRS prior to their recruitment to this study (range 2–5). Sixty percent (*n* = 48) showed endoscopic signs of disease recurrence at Visit 2. Again, the presence of *S. aureus* postoperatively was significantly associated with failure. Having seasonal allergies was a minor statistically significant protective factor in this subgroup of patients. There were no significantly differences in the various laboratory values evaluated.

**Table 3:** Disease remission vs. recurrence in revision ESS patients.

**Total: 80 patients**	**Remission**	**Recurrence**	***p*-value**
Patients	32 (40%)	48 (60%)	
Age	52.9	50.0	*p* = 0.295
Male	16 (50%)	26 (54.2%)	*p* = 0.715
Female	16 (50%)	22 (45.8%)	
Asthma	22 (68.8%)	37 (77.1%)	*p* = 0.407
Smoking			
Never	12 (37.5%)	22 (45.8%)	*p* = 0.485
Active	8 (25.0%)	7 (14.6%)	
Former	12 (37.5%)	19 (39.6%)	
Polyposis	31 (96.9%)	42 (87.5%)	*p* = 0.233
Race			
Caucasian	28 (87.5%)	44 (91.7%)	*p* = 0.321
Hispanic	1 (3.1%)	3 (6.3%)	
Asian	1 (3.1%)	1 (2.1%)	
Arabic	2 (6.3%)	0 (0.0%)	
Allergies (all types)	28 (87.5%)	35 (72.9%)	*p* = 0.118
Seasonal	14 (43.8%)	11 (22.9%)	*p = 0.049*
NSAID	4 (12.5%)	16 (33.3%)	*p = 0.039*
*S. aureus* (pre-ESS)	3 (9.7%)	12 (25%)	*p* = 0.141
*S. aureus* (post-ESS)	3 (10.0%)	20 (48.8%)	*p < 0.001*
*P. aeruginosa* (pre-ESS)	1 (3.2%)	5 (10.4%)	*p* = 0.395
*P. aeruginosa* (post-ESS)	0 (0.0%)	5 (12.2%)	*p = 0.069*
Gram negative (pre-ESS)	7 (22.6%)	11 (22.9%)	*p* = 1.000
Gram negative (post-ESS)	5 (16.7%)	10 (24.4%)	*p* = 0.560
SNSS (post-ESS)	4.8	5.3	*p* = 0.660
SNOT22 (post-ESS)	29.2	31.9	*p* = 0.619
Lund-Kennedy (post-ESS)	1.0	6.1	*p < 0.001*

## Discussion

Refractory CRS unresponsive to maximal medical and surgical therapy remains an important and growing burden and may even be underestimated in certain subgroups. In this study, we evaluated the outcome of primary or revision ESS followed by 4 months of budesonide nasal irrigations (BUD) in a subgroup of patients deemed at a high risk of disease recurrence after ESS. In previous retrospective reports from our group, we had reported a recurrence rate slightly over 30% (Maniakas and Desrosiers, 2014). In this prospective trial, using well-documented rigorous endoscopic assessment of mucosal disease, failure rates are seen to be higher, attaining 50% in this high risk patient population.

Patients with previous ESS were at the highest risk of treatment failure. This is of particular interest as more than 80% of patients who failed ESS+BUD had had at least one ESS prior to enrollment. This remains in line with the current literature that revision ESS is a poor prognostic factor (Lee et al., 2008; Philpott et al., 2015). The burden of revision ESS is also depicted in the frequency of procedures one patient may undergo in an attempt to control the disease. In this study, of the 80 patients having had prior ESS, close to 45% had undergone more than 1 and as many as 5 procedures. This rate is comparable to some recent studies which demonstrate a similar problematic (Philpott et al., 2015). Patients are therefore put at risk to the various intra and postoperative complications associated with ESS, which are far from negligible and are known to significantly increase in frequency in previously operated sinonasal cavities. What remains certain is the need for additional clinically relevant therapeutic options that can offer a long-term alternate solution to revision ESS.

The most interesting finding in our prospective study was the contribution of microbes to early disease recurrence. Colonisation with *S. aureus* was present in nearly half of all patients having failed ESS+BUD at 4 months, compared to only 13% of patients with disease remission. This finding is in line with the current literature where a positive culture for *S. aureus* is described as a poor prognostic factor post-ESS (Jervis-Bardy et al., 2011). Interestingly, when present preoperatively, *S. aureus* was cleared in 75% of patients with disease remission, but only 21% cleared it in the group with disease recurrence.

A role for *S. aureus* in disease development or persistence has been long suspected. In an earlier publication, we showed that recovery of *S aureus* is higher in diseased post-ESS cavities than in healthy ones (Al-Shemari et al., 2007). This may represent isolate-specific behavior in *S. aureus*. Our group has previously demonstrated that biofilm-forming capacity by *S. aureus* was associated with worse outcome (Bendouah et al., 2006), and Tan et al. have demonstrated that the presence of intraepithelial forms of small-colony variant *S. aureus* was associated with a negative outcome (Tan et al., 2014), possibly via reduction of immunity (Ou et al., 2016).

While the mechanisms for this have been postulated to include (i) modulation of the immune system via enterotoxin-mediated super antigen activation of the immune system and (ii) bacterial biofilm formation by *S. aureus*, recent evidence suggest that *S. aureus* may also employ other strategies, rendering it a realistic marker of disease development and a target for therapy. In addition to already described virulence strategies for *S. aureus*, our group has recently suggested two novel ones: (i) An immunomodulatory effect, modulated via induction of excessively high levels of IL-10, an anti-inflammatory cytokine (Chau et al., 2009; Schwartz et al., 2017), and (ii) a deleterious effect on *in-vitro* models of epithelial regeneration and wound repair, particularly in cell cultures raised form CRS patients (Valera et al., in press).

What remains to be determined, and where research groups are focusing significant time, is the origin of the mucosal appearance of this pathogen, either through neo-colonization from the external environment or the nasal vestibule introduced into the ethmoid cavities at the time of surgery or via planktonic dissemination following surgical disruption of an existing *S. aureus* biofilm (Jervis-Bardy et al., 2011). This does not appear to be influenced by the use of post-operative antibiotics: in a prospective trial comparing post-operative use of Chinese herbal medicine, amoxicillin, and placebo following ESS, colonization with *S. aureus* was present to an equal level in all three groups, with approximately one-half of all post-ESS patients showing evidence of neo-colonization with *S. aureus* (Liang et al., 2011). Additionally, the question of whether some individuals are genetically predisposed to be susceptible to *S. aureus* colonization. A previous pooling-based genome wide association scan (pGWAS) (Cormier et al., 2014) has suggested that variations in candidate genes implicated in bacterial engulfment and destruction as well those impacting epithelial barrier function are associated with *S. aureus* carriage in CRS patients, suggesting a potential genetically-determined basis to susceptibility.

Asthma with nasal polyposis and intolerance to non-steroidal anti-inflammatories, also known as aspirin exacerbated respiratory disease (AERD), was also a significant association to ESS+BUD failure. Of 17 patients with this diagnosis, 13 (76.5%) had disease recurrence. These findings are concordant with the current literature on this disease and complement the findings of Mendelsohn et al. (Nader et al., 2010) that demonstrated increased rates of disease recurrence and revision ESS.

Interestingly, although more elevated in the group with disease recurrence, SNSS and SNOT-22 scores were not statistically significantly different between the two groups at either time-point. Our findings did demonstrate a strong correlation between the two scores and further strengthened the comparability of the SNSS to the SNOT-22 score in evaluating active disease. However, SNOT-22 scores showed only a small correlation, although significant, to a poor Lund-Kennedy score. This discrepancy is possibly due to the self-reporting bias on subjective measures. The significantly higher Lund-Kennedy scores observed in the disease-recurrence groups despite similar symptom intensity, emphasizing the importance of considering objective outcome in addition to patient-reported ones.

A potential limitation of our study can be argued to that 4 months may be considered as a short follow up period after ESS. However, several authors have previously shown that endoscopic signs of recurrence are seen as early as 4 months (Stjärne et al., 2009; Amali et al., 2015) and that these appear stable over an additional 12 month follow up period (DeConde and Soler, 2016). Furthermore, data on SNSS and SNOT-22 are self-reported and patients may either not accurately recollect the severity or prevalence of their symptoms leading to a recollection bias, it can also lead to a response bias due to personal perceptive influences on one's symptomatology. It is for these exact reasons that studies using subjective questionnaires require an objective measurable value, such as the Lund-Kennedy endoscopic score.

## Conclusion

Patients undergoing revision ESS are at high risk of disease recurrence, even when budesonide irrigations are used postoperatively. Presence of *S. aureus* on culture pre-operatively or at 4 months post-ESS is associated with a negative outcome. This suggests that *S. aureus* negatively influences outcome, possibly via a number of potential interactions with the (i) immune system, (ii) regeneration and repair of the sinus epithelium, or (iii) via interference with the sinus microbiome. This suggests that *S. aureus* may be a simple and inexpensive biomarker for disease severity and indicates a clear need to better appreciate *S. aureus'* mechanistic contribution to disease development and persistence in order to develop targeted therapeutic strategies. Additional studies on microbiome and sinus mucosal gene expression will help in the understanding of several persisting pathophysiologic queries.

## Author contributions

AM: study design; data collection and analysis; manuscript drafting, reviewing, final approval, accountability for all aspects of the work; M-HA: data collection and analysis; manuscript reviewing, final approval, accountability for all aspects of the work; AR and SA: data collection; manuscript reviewing, final approval, accountability for all aspects of the work; SN: data collection; manuscript reviewing; final approval, accountability for all aspects of the work; LM: data collection and analysis; manuscript drafting; final approval, accountability for all aspects of the work; MD: study design; data analysis; manuscript writing, reviewing, final approval, accountability for all aspects of the work.

### Conflict of interest statement

The authors declare that the research was conducted in the absence of any commercial or financial relationships that could be construed as a potential conflict of interest.

## References

[B1] Al-Shemari H. Abou-Hamad W. Libman M. Desrosiers M. (2007). Bacteriology of the sinus cavities of asymptomatic individuals after endoscopic sinus surgery. J. Otolaryngol. 36, 43–48. 10.2310/7070.2006.0019 17376350

[B2] Amali A. Saedi B. Rahavi-Ezabadi S. Ghazavi H. Hassanpoor N. (2015). Long-term postoperative azithromycin in patients with chronic rhinosinusitis: a randomized clinical trial. Am. J. Rhinol. Allergy 29, 421–424. 10.2500/ajra.2015.29.4244 26637580

[B3] Anand V. K. (2004). Epidemiology and economic impact of rhinosinusitis. Ann. Otol. Rhinol. Laryngol. Suppl. 193, 3–5. 10.1177/00034894041130S502 15174752

[B4] Bendouah Z. Barbeau J. Hamad W. Desrosiers M. (2006). Biofilm formation by *Staphylococcus aureus* and *Pseudomonas aeruginosa* is associated with an unfavourable evolution after surgery for chronic sinusitis and nasal polyposis. Otolaryngol. Head Neck Surg. 134, 991–996. 10.1016/j.otohns.2006.03.001 16730544

[B5] Browne J. P. Hopkins C. Slack R. Cano S. J. (2007). The Sino-Nasal Outcome Test (SNOT): can we make it more clinically meaningful? Otolaryngol. Head Neck Surg. 136, 736–741. 10.1016/j.otohns.2007.01.024 17478207

[B6] Chau T. A. McCully M. L. Brintnell W.. (2009). Toll-like receptor 2 ligands on the staphylococcal cell wall downregulate superantigen-induced T cell activation and prevent toxic shock syndrome. Nat. Med. 15, 641–648. 10.1038/nm.1965 19465927

[B7] Chen Y. Dales R. Lin M. (2003). The epidemiology of chronic rhinosinusitis in Canadians. Laryngoscope 113, 1199–1205. 10.1097/00005537-200307000-00016 12838019

[B8] Cormier C. Mfuna Endam L. Filali-Mouhim A. Boisvert P. Boulet L. P. Boulay M. E.. (2014). A pooling-based genomewide association study identifies genetic variants associated with *Staphylococcus aureus* colonization in chronic rhinosinusitis patients. Int. Forum Allergy Rhinol. 4, 207–215. 10.1002/alr.21276 24431132

[B9] DeConde A. S. Soler Z. M. (2016). Chronic rhinosinusitis: Epidemiology and burden of disease. Am. J. Rhinol. Allergy 30, 134–139. 10.2500/ajra.2016.30.4297 26980394

[B10] Hastan D. Fokkens W. J. Bachert C. Newson R. B. Bislimovska J. Bockelbrink A. (2011). Chronic rhinosinusitis in Europe–an underestimated disease. A GA(2)LEN study. Allergy 66, 1216–1223. 10.1111/j.1398-9995.2011.02646.x 21605125

[B11] Jackson D. J. Gern J. E. Lemanske R. F. Jr. (2017). Lessons learned from birth cohort studies conducted in diverse environments. J. Allergy Clin. Immunol. 139, 379–386. 10.1016/j.jaci.2016.12.941 28183432 PMC5508737

[B12] Jervis-Bardy J. Foreman A. Boase S. Valentine R. Wormald P.-J. (2011). What is the origin of *Staphylococcus aureus* in the early postoperative sinonasal cavity? Int. Forum Allergy Rhinol. 1, 308–312. 10.1002/alr.20050 22287437

[B13] Lee J. Y. Lee S. W. Lee J. D. (2008). Comparison of the surgical outcome between primary and revision endoscopic sinus surgery for chronic rhinosinusitis with nasal polyposis. Am. J. Otolaryngol. 29, 379–384. 10.1016/j.amjoto.2007.11.005 19144298

[B14] Psaltis A. J. Li G. Vaezeafshar R. Cho K. S. Hwang P. H. (2014). Modification of the Lund-Kennedy endoscopic scoring system improves its reliability and correlation with patient-reported outcome measures. Laryngoscope 124, 2216–2223. 10.1002/lary.24654 24615873

[B15] Liang K. L. Su Y. C. Tsai C. C. Lin J. S. Jiang R. S. Su M. C. (2011). Postoperative care with Chinese herbal medicine or amoxicillin after functional endoscopic sinus surgery: a randomized, double-blind, placebo-controlled study. Am. J. Rhinol. Allergy 25, 170–175. 10.2500/ajra.2011.25.3610 21679528

[B16] Maniakas A. Desrosiers M. (2014). Azithromycin add-on therapy in high-risk postendoscopic sinus surgery patients failing corticosteroid irrigations: a clinical practice audit. Am. J. Rhinol. Allergy 28, 151–155. 10.2500/ajra.2013.27.4017 24598145

[B17] Mendelsohn D. Jeremic G. Wright E. D. Rotenberg B. W. (2011). Revision rates after endoscopic sinus surgery: a recurrence analysis. Ann. Otol. Rhinol. Laryngol. 120, 162–166. 10.1177/000348941112000304 21510141

[B18] Nader M.-E. Abou-Jaoude P. Cabaluna M. Desrosiers M. (2010). Using response to a standardized treatment to identify phenotypes for genetic studies of chronic rhinosinusitis. J. Otolaryngol. Head Neck Surg. 39, 69–75. 20122348

[B19] Ou J. J. Drilling A. J. Cooksley C.. (2016). Reduced innate immune response to a *Staphylococcus aureus* small colony variant compared to its wild-type parent strain. Front. Cell. Infect. Microbiol. 6:187. 10.3389/fcimb.2016.00187 28083514 PMC5183720

[B20] Philpott C. Hopkins C. Erskine S. Kumar N. Robertson A. Farboud A.. (2015). The burden of revision sinonasal surgery in the UK-data from the Chronic Rhinosinusitis Epidemiology Study (CRES): a cross-sectional study. BMJ Open 5:e006680. 10.1136/bmjopen-2014-006680 25926143 PMC4420947

[B21] Piccirillo J. F. Merritt M. G. Jr. Richards M. L. (2002). Psychometric and clinimetric validity of the 20-Item Sino-Nasal Outcome Test (SNOT-20). Otolaryngol. Head Neck Surg. 126, 41–47. 10.1067/mhn.2002.121022 11821764

[B22] Rudmik L. Smith T. L. Schlosser R. J. Hwang P. H. Mace J. C. Soler Z. M. (2014). Productivity costs in patients with refractory chronic rhinosinusitis. Laryngoscope 124, 2007–2012. 10.1002/lary.24630 24619604 PMC4125547

[B23] Schwartz J. S. Al-Mot S. Endam M. F. Alromaih S. Madrenas J. Desrosiers M. (2017). Bacterial immune evasion via an IL-10 mediated host response, a novel pathophysiologic mechanism for chronic rhinosinusitis. Rhinology 155, 227–233. 10.4193/Rhin16.199 28315920

[B24] Stjärne P. Olsson P. Alenius M. (2009). Use of mometasone furoate to prevent polyp relapse after endoscopic sinus surgery. Arch. Otolaryngol. Head Neck Surg. 135, 296–302. 10.1001/archoto.2009.2 19289710

[B25] Tan N. C. Cooksley C. M. Roscioli E. Drilling A. J. Douglas R. Wormald P. J.. (2014). Small-colony variants and phenotype switching of intracellular *Staphylococcus aureus* in chronic rhinosinusitis. Allergy 69, 1364–1371. 10.1111/all.12457 24922342

[B26] Valera F. Ruffin M. Adam D. Maillé E. Ibrahim B. Berubé J. (in press). *Staphylococcus aureus* impairs sinonasal epithelial repair: effects in CRSwNP control subjects. J. Allergy Clinical Immunol. 10.1016/j.jaci.2018.05.035 29935218

